# UBA3 promotes the occurrence and metastasis of intrahepatic cholangiocarcinoma through MAPK signaling pathway: UBA3 promotes the occurrence and metastasis of ICC

**DOI:** 10.3724/abbs.2024014

**Published:** 2024-01-31

**Authors:** Huhu Zhang, Jiahua Yang, Qinghang Song, Xiaoyan Ding, Fulin Sun, Lina Yang

**Affiliations:** 1 Department of Genetics and Cell Biology Basic Medical College Qingdao University Qingdao 266071 China; 2 School of Basic Medicine Qingdao University Qingdao 266071 China; 3 Institute of Brain Science and Disease Shandong Provincial Key Laboratory of Pathogenesis and Prevention of Neurological Disorders Qingdao University Qingdao 266071 China; 4 College of Medicine Qingdao University Qingdao 266071 China

**Keywords:** intrahepatic cholangiocarcinoma, UBA3, ANXA2, bufalin, prognosis, proliferation, migration

## Abstract

Intrahepatic cholangiocarcinoma (ICC) accounts for approximately 15% of primary liver cancers, and the incidence rate has been increasing in recent years. Surgical resection is the best treatment for ICC, but the 5-year survival rate is less than 30%. ICC signature genes are crucial for the early diagnosis of ICC, so it is especially important to identify signature genes. The aim of this study is to screen the signature genes of ICC and find the potential target for the treatment of ICC. We find that UBA3 is highly expressed in ICC, and knockdown of *UBA3* inhibits ICC proliferation, invasion and migration. Mechanistic experiments show that UBA3 promotes ICC proliferation, invasion and migration by affecting ANXA2 through the MAPK signaling pathway. UBA3 is a target of bufalin, and bufalin targeting UBA3 inhibits ICC development and progression through the MAPK signaling pathway. In conclusion, our study shows that bufalin inhibits ICC by targeting UBA3, which has emerged as a new biomarker and potential therapeutic target for ICC.

## Introduction

Intrahepatic cholangiocarcinoma (ICC) is the second most common primary liver cancer after hepatocellular carcinoma (HCC), and its incidence and mortality rates are increasing worldwide [ 1‒ 3]. ICC is a tumor of the bile ducts within the liver that arises primarily from the uncontrolled proliferation of transformed cholangiocytes [ 4, 5]. ICC usually presents as an intrahepatic mass lesion, and no solid mass lesion is found on cross-sectional images [6]. ICC has no obvious clinical symptoms in the early stage, and most patients have lost the opportunity to undergo surgery when diagnosed, which is associated with a poor prognosis [7]. Therefore, more diagnostic tools for identifying early biomarkers are needed.

UBA3 has been implicated in various biological processes and diseases. For example, UBA3 is induced in differentiated preadipocytes and regulates adipogenesis and lipid droplet formation [8]. UBA3 also modulates oxidative phosphorylation and the tricarboxylic acid cycle in breast cancer cells by affecting the neddylation of mitochondrial proteins, and the activity of the PI3K/AKT signaling pathway is positively correlated with the expression of UBA3 [9]. Moreover, UBA3 expression is influenced by glucose level and epigenetic modifications, and UBA3 mediates the neddylation of *PTEN*, a tumor suppressor gene, in breast cancer [ 10 , 11]. In addition, UBA3 has been reported to be involved in liver cancer. A study showed that UBA3 deficiency caused fatty liver and hepatocyte damage in mice [8]. Another study revealed that UBA3 was overexpressed in hepatocellular carcinoma tissues and cell lines and that its knockdown inhibited cell proliferation and induced apoptosis [12] .

UBA3 has also emerged as a potential drug target for cancer therapy. MLN4924 is a selective inhibitor of NAE that covalently binds to the cysteine residue of UBA3 and blocks its interaction with NEDD8 [ 13‒ 15]. MLN4924 has displayed preclinical anti-tumor activity *in vitro* and *in vivo* against various types of cancers, including leukemia, lymphoma, multiple myeloma, glioblastoma, ovarian cancer, pancreatic cancer, prostate cancer, and lung cancer. MLN4924 exerts its anti-cancer effects by disrupting neddylation-dependent processes such as proteasome function, DNA replication, the DNA damage response, cell cycle progression, apoptosis, autophagy, and signal transduction. MLN4924 has also shown promising clinical activity in patients with refractory hematologic malignancies. However, studies focused on the role of UBA3 in ICC are lacking.

MAPK is an intracellular class of serine/threonine protein kinases that play a key role in cell proliferation and growth [ 16, 17]. MAPK signaling pathway accelerates cell division and proliferation by increasing the expression of CyclinD1, which promotes the transition from G1 to S phase of the cell cycle [18]. Phospholipase A2 promotes the proliferation and growth of oesophageal adenocarcinoma through the activity of ERK1/2, and the use of ERK1/2 can inhibit the growth of oesophageal adenocarcinoma [19]. In addition, VEGF can reduce adhesion between tumor cells and promote tumor infiltration and metastasis through phosphorylation of MAPK [20]. An in-depth understanding of the relationship between the MAPK signaling pathway and ICC provides a theoretical basis for the exploration of targets in ICC and new ideas for the development of clinical drugs.

In this study, we used multiple bioinformatics approaches to determine whether *UBA3* is a signature gene for ICC diagnosis. We also evaluated the prognostic value of UBA3 expression in ICC patients. We further investigated the role of UBA3 in ICC cell proliferation and migration, as well as its underlying mechanism. We identified ANXA2 as a downstream molecule of UBA3 that mediates its effects on ICC cell behavior. We also screened bufalin as a candidate inhibitor of UBA3 by molecular docking and validated its anti-ICC activity *in vitro*. Our study provides new insights into the biology and functions of UBA3 in ICC and suggests novel strategies for ICC diagnosis and treatment.

## Methods and Materials

### Data sources

In the present study, the GSE33327 dataset was downloaded from the Gene Expression Omnibus (GEO) database ( http://www.ncbi.nlm.nih.gov/geo/). As a training set, GSE33327 included 149 ICC patients and 6 normal controls. The UALCAN website ( https://ualcan.path.uab.edu/analysis.html) was subsequently used to reverse-validate the ICC data from the TCGA database.

### Identification of DEGs

Using the LIMMA package of R software [21], differentially expressed genes (DEGs) between the ICC patient cohort and control cohort were analyzed with the following criteria: *P<*0.05 and |fold change (FC)|>0.25. Volcano plots were generated to show the DEGs, while heatmaps were generated to show the top 30 up- and down-regulated DEGs.

### Functional and pathway enrichment analyses

The clusterProfiler package in R was used to carry out functional enrichment analysis of the DEGs via Gene Ontology (GO) and Kyoto Encyclopedia of Genes and Genome (KEGG) analyses [22]. Three types of processes, namely, biological process (BP), cell composition (CC) and molecular function (MF), were analyzed and determined, which is helpful for exploring the biological process determination of differential genes. KEGG analysis was used to explore potential signal pathways.

### Weighted gene co-expression network analysis

Analysis of co-expression networks in the GSE33327 cohort using the WGCNA network, with co-expression network analysis constructed from weighted genes [23]. The PickSoftThreshold function of WGCNA was used to calculate the soft threshold power as well as the adjacency values. Subsequently, the adjacency matrix was converted to a topological overlap matrix (TOM), and the corresponding dissimilarity was calculated to perform a hierarchical clustering analysis. Co-expressed gene modules were identified using a dynamic tree cutting method with a minimum module size of 50. The gene modules were subsequently linked to the ICC as indicated by the gene significance (GS) values and module affiliation (MM) values, after which major modules were ultimately identified.

### Signature gene identification

The candidate hub genes were identified by the intersection of DEGs with key module genes. Subsequently, three machine learning algorithms, minimum absolute shrinkage selection (SVM-RFE), LASSO and random forest, were used to screen for hub genes. The support vector machine (SVM), which is a surveillance machine learning method with support vectors, searches for the best variables by removing the feature vectors generated by the support vector machine [24]. Analysis of the selected biomarkers in the ICC diagnosis was performed using the SVM classifier in the R package e1071 for classification; K=5 was the setting for K-fold cross-validation, and the parameter for the above pair of halves was determined to be 100.

LASSO analysis was performed using the “glmnet” package with penalized parameters for 10-fold crossing validation [25]. In addition, the classification of the DEGs among the hub genes was performed by applying the R package ″random forest″ [26]. The independent random forest model was used to determine the optimal number of variables by calculating the average error rate of the candidate hub genes. The intersecting genes of these three machine learning algorithms were used as the ICC signature genes. The diagnostic efficiency of these signature genes was assessed using the area under the curve (AUC) of the receptor operating characteristic curve (ROC). An AUC above 0.7 indicated a good diagnosis.

### Gene set enrichment analysis

To define the relationship between signature genes and signaling pathways, we grouped the ICC cohort according to median hub gene expression and performed gene set enrichment analysis (GSEA) on different subgroups adjusted for *P*<0.05 [27].

### Cell culture

The human ICC cell lines HCCC-9810 (CVCL_6908), RBE (CVCL_4896), QBC-939 (CVCL_6942) and HuCC-T1 (CVCL_0324) were obtained from Procell (Wuhan, China). All cells were cultured at 37°C in a constant temperature incubator in RPMI 1640 medium (Gibco, Carlsbad, USA) supplemented with 10% serum.

### Cell transfection

After HCCC-9810 cells were seeded and cultured overnight, siUBA3 and siANXA2 were transfected using siRNA mate (GenePharma, Shanghai, China) according to the manufacturer’s instructions. siRNA transfection complexes were prepared with siRNA mate in opti-MEM (Thermo Fisher, Waltham, USA) and after 48 h of transfection, cells were lysed. Silenced RNA (siRNA) vectors targeting *UBA3* and *ANXA2* were constructed by OBiO Technology Company (Shanghai, China). The RNAi target sequences are shown below: UBA3-RNAi, 5′-CCUCUAUUGAAGAACGAACAATT-3′; ANXA2-RNAi, 5′-TGTGTGGTGGAGATGACTGA-3′, and control-RNAi, 5′-UUCUCCGAACGUGUCACGUTT-3′.

### Cell viability assay

Cell viability was detected by cell counting kit-8 (CCK8) assay. HCCC-9810 cells were cultured in 96-well plates overnight. After 24, 48 and 72 h of bufalin treatment, 10 μL of CCK8 solution (Beyotime Biotechnology, Shanghai, China) was added to the culture and incubated for 1.5 h, and then the OD value at 450 nm was measured.

### Wound healing assay

Cell migration activity was measured by wound healing assay. Two to three horizontal lines were evenly drawn on the back of the 6-well plate, and 2 mL HCCC-9810 cells at cell density of 5.0×10 ^5^/mL were added. The cells were cultured for 24 h. When the cell density reached 95%, the cell layers were scratched along the underside with a 200-μL tip and washed with PBS buffer for 2–3 times to remove the cell debris. The culture was continued and photographs were taken at 24 h and 48 h respectively.

### Transwell assay

For cell migration assay, transwell inserts with 6.5-mm polycarbonate membrane and 8.0-μm pore were used. HCCC-9810 cells were suspended in serum-free medium and counted. After centrifugation, the cells were resuspended and 200 μL of cell suspension was inoculated in the upper chamber. Then, 600 μL of medium containing 20% fetal bovine serum was added to the lower chamber. After 48 h of culture, the transwell chambers were removed and the supernatants were aspirated. The migrated cells were fixed with 4% polyformaldehyde for 30 min and stained with 0.1% crystal violet for 15 min, after which the average cell number per well was calculated with ImageJ (NIH, Bethesda, USA).

### Proteome microarray

The preparation of human protein microarrays and the synthesis of biotin-bufalin were carried out as previously described [ 28, 29]. The samples were sealed in buffer [1% bovine serum albumin (BSA) and 0.1% Tween 20 (TBST)] and stirred gently at 25°C for 1 h. Biotin-bufalin was diluted to 10 μM in blocking buffer and incubated at 25°C for 1 h on a proteome microarray. The biotin-bufalin was washed with thiobarbituric acid for 3 times (5 min each), and then incubated with Cy3-streptavidin (Smart-Lifesciences, Changzhou, China) at 1:1000 for 1 h at 25°C, followed by washing with thiobarbituric acid for 3 times (5 min each). The microarrays were dried at 250 *g* for 3 min and scanned with a GenePix 4200A microarray scanner (Molecular Devices, San Jose, USA), after which the results were recorded. GenePix Pro-6.0 software was used for data analysis.

### Molecular docking

To evaluate the binding energy and interaction pattern of the candidate bufalin agents with UBA3, we used AutodockVina 1.2.2, a computerized protein–ligand docking software [30]. The molecular structure of bufalin was obtained from the PubChem compound database ( https://pubchem.ncbi.nlm.nih.gov/) [31]. We first prepared the protein and ligand files by converting all the protein and molecule files to PDBQT format, removing all water molecules, and adding polar hydrogen atoms. The grid boxes were centered to cover the structural domains of each protein and to accommodate free molecular motion.

### Western blot analysis

Total protein extracts from HCCC-9810, RBE, QBC-939 and HuCC-T1 cells were separated by 10% sodium dodecyl sulfate-polyacrylamide gel electrophoresis (SDS-PAGE) and transferred to polyvinylidene fluoride (PVDF) membranes. After being blocked with 5% solution of skim milk powder and TBST, the membranes were incubated overnight with primary antibodies against UBA3, ANXA2, N-Cadherin, Vimentin, MEK, ERK, ^Ser217/221^P-MEK ^Thr158/Tyr187^P-ERK or GAPDH (Abmart, Shanghai, China), followed by incubation with the secondary antibody (ABMT-PT; Abmart) for 1.5 h at room temperature. Finally, protein bands were visualized with P-ECL luminescent solution (Epizyme Biomedical Technology, Shanghai, China) and band density was analyzed using ImageJ. GAPDH was used as the loading control.

### Immunohistochemistry (IHC) assay

Fifty postoperative specimens of intrahepatic cholangiocarcinoma and 50 tissue specimens of normal liver tissue were collected from the Affiliated Hospital of Qingdao University from 2019 to 2022. All human experiments were conducted in compliance with the ethical guidelines established by the Institutional Ethical Committee of Qingdao University (Approval No. QDU-HEC-2022289).

IHC was performed on 4-μm-thick FFPE sections using the Dako EnVision Plus kit (Dako, Glostrup, Denmark) according to the manufacturer’s instructions. Slides were deparaffinized and pretreated with 1 mM EDTA and heat-mediated antigen retrieval solution in a microwave oven. Further steps were done at room temperature in a hydrated chamber. Slides were preincubated in 20% normal goat serum and then with anti-UBA3 rabbit monoclonal antibody (1:200; Abmart). After being washed with PBS, the slides were incubated with horseradish peroxidase-conjugated anti-rabbit IgG secondary antibody. All slides were counterstained with hematoxylin. The slices were then dehydrated in alcohol, transparent in xylene and sealed with neutral resin. Data acquisition was performed under an inverted fluorescence microscope (Nikon, Tokyo, Japan).

### Pull-down assay

HCCC-9810 cells were seeded in a 15-cm culture dish and cultured overnight in CO _2_ incubator at 37°C. HCCC-9810 cells were collected and lysed with RIPA Lysis Buffer (Epizyme Biomedical Technology). After incubation with bufalin (Selleck, Shanghai, China), the extract was bound to streptavidin coupled magnetic beads (Smart-Lifesciences) and incubated at 4°C for 1‒2 h, and the nonspecific binding protein molecules were washed away with Pre-cooled PBS. The protein products obtained by washing and elution were detected by SDS-PAGE.

### Statistical analysis

The flow chart for this study is shown in Supplementary Figure S1. Data are shown as the mean±SD. All the bioinformatics statistical analyses for this study were performed using R software (version 4.2.2). All the statistical analyses were performed using GraphPad Prism 8 and ImageJ unless otherwise stated. *P<*0.05 was considered statistically significant.

## Results

### DEGs between ICC patients and controls identified by differential gene expression analysis and functional enrichment analysis

**Figure 1:**
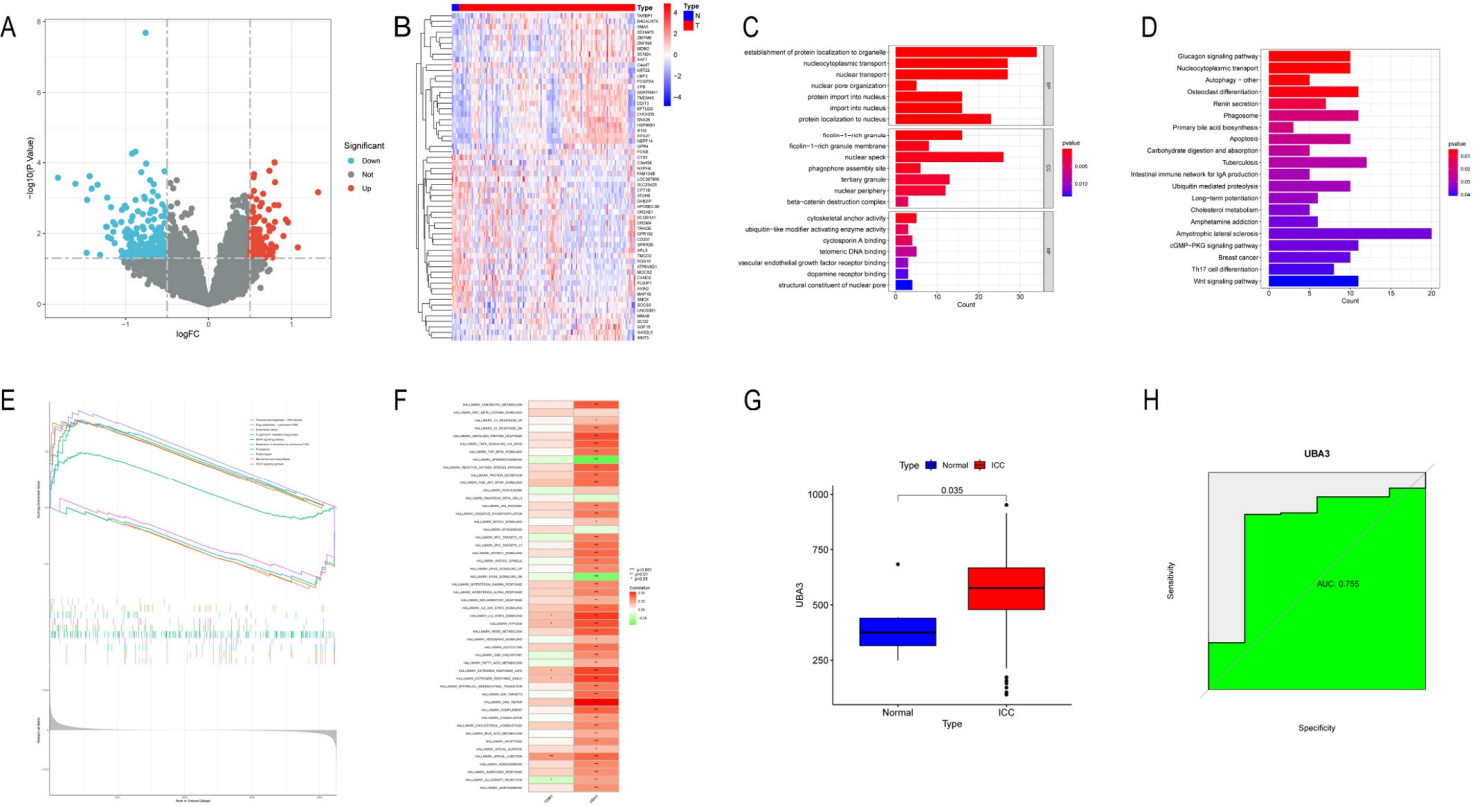
Figure 1 *UBA3* is a signature gene of ICC identified by bioinformatics analysis (A) The expression of DEGs in the ICC and healthy populations is shown in the volcano plot. (B) Heatmap showing the top 30 up-regulated and 30 down-regulated DEGs. (C) BP, CC and MF analysis of the top 10 functionally enriched genes. (D) KEGG analysis of the DEGs. (E) GSEA of UAB3 in ICC. (F) Correlation chart of two signature genes with 50 GSEA hallmark gene sets. (G) UBA3 expression between ICC patients and healthy individuals. (H) Diagnostic performance of UBA3 as shown by the ROC curve.

The data from the ICC patients and healthy controls were analyzed using the ″limma″ package. A total of 781 DEGs were screened, including 473 up-regulated genes and 308 down-regulated genes ( Figure 1A). A heatmap revealed the top 30 DEGs that were up- and down-regulated between the ICC patients and the healthy individuals ( Figure 1B). GO analysis of the 781 potentially significant genes was performed using the DAVID database. The GO analysis included three categories: BP, CC and MF ( Figure 1C). KEGG analysis revealed that the three pathways associated with the genes exhibiting the most enrichment are involved in the glucagon signaling pathway, nucleocytoplasmic transport, and autophagy. In addition, the Wnt signaling pathway was also enriched ( Figure 1D). Magenta and Midnightbluem modules are key modules relevant to patients with ICC ( Supplementary Figure S2). LASSO, SVM-RFE and Random Forest Algorithms were used to screen the signature genes of intrahepatic cholangiocarcinoma. Two ICC signature genes were finally identified, including *TOM1* and *UBA3* ( Table 1 and Supplementary Figure S3).

**Table:** **Table 1** Key genes screened by LASSO, SVM-RFE and random forest analysis

Machine learning algorithm	LASSO	SVM-RFE	Random forest
Gene	DPRXP4, UBA3, C19orf56, ALDH1A3, TOM1, ACTR2, GABPB2, RPS28, SLC22A7, LEAP2	DPRXP4, UBA3, LEAP2, C19orf56, GABPB2, PPA2, TOM1, TM4SF1	UBA3, TOM1, ACTR2, CALM3, ZNF358, ZMYM6, TM4SF1, ALDH1A3, RPS28, TTC21B

### Pathway insights through GSEA analysis

GSEA analysis was used to assess the signaling pathways associated with the signature genes. The first 10 signaling pathways are shown in Supplementary Figure S4. The results revealed that TOM1 is strongly associated with base excision repair, the cell cycle, focal adhesion, glycine, serine and threonine metabolism, human papillomavirus infection, legionellosis, leishmaniasis, long-term potentiation, protein processing in the endoplasmic reticulum, and the viral life cycle-HIV-1 ( Supplementary Figure S4A). The expression of UBA3 is significantly correlated with chemical carcinogenesis-DNA adducts, drug metabolism-cytochrome P450, endometrial cancer, Fc gamma R-mediated phagocytosis, the MAPK signaling pathway, metabolism of xenobiotics by cytochrome P450, the proteasome, protein export, steroid hormone biosynthesis, and the VEGF signaling pathway ( Figure 1E). In summary, both TOM1 and UBA3 are associated with signaling pathways involved in the development, invasion and metastasis of ICC. In fact, there is a significant positive correlation (0.38) between TOM1 and UBA3. Furthermore, 50 signaling pathways were analyzed, among which TOM1 and UBA3 were more important for cancer proliferation, invasion and metastasis. UBA3 plays a critical role in ICC proliferation, invasion and metastasis relative to TOM1 ( Figure 1F and Supplementary Figure S4B).

### Diagnostic potential of characteristic genes in ICC prediction

The expressions of the selected characteristic genes in patients with ICC were greater than those in the normal group, which indicates that TOM1 ( Supplementary Figure S4C) and UBA3 ( Figure 1G) may play a potential role in the occurrence and development of ICC. In addition, the area under the curve (AUC) of the receiver operating characteristic (ROC) curve for these characteristic genes was 0.755 for UBA3, *P*<0.05 ( Figure 1H); 0.725 for TOM1, *P*>0.05 (rank sum test); thus, TOM1 was excluded ( Supplementary Figure S4D). We also used UALCAN software to analyze the TCGA database and evaluate the ability of the *UBA3* gene to predict ICC. A significant correlation was found between the expression of UBA3 and the degree of malignancy of ICC ( Supplementary Figure S5). These findings indicate that the selected characteristic genes have significant diagnostic value in predicting ICC.

### UBA3 shows higher expression in ICC cells and tissues

**Figure 2:**
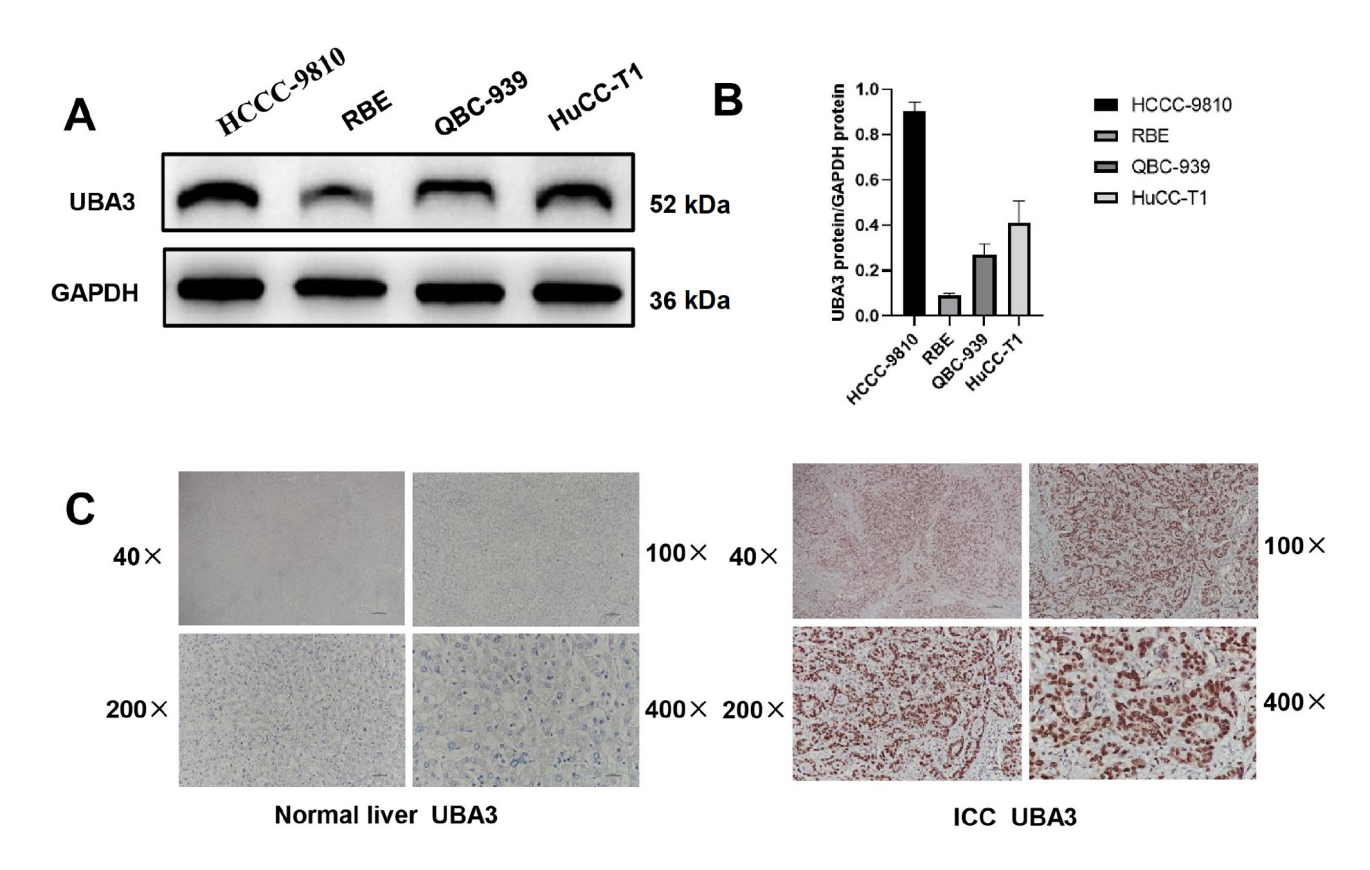
Figure 2 UBA3 showed higher expression in ICC cells and tissues (A,B) Verification of UBA3 protein expression levels in four ICC cell lines. (C) IHC results for clinical samples of normal liver and ICC tissues.

By investigating the functional implications of UBA3 in the cellular processes of ICC, we aimed to explore its potential roles. Previous study has reported increased UBA3 expression in ICC compared to normal tissue; however a comprehensive exploration of this topic is lacking [32]. Therefore, we performed a comparative analysis of UBA3 expression in four different types of ICC cells. Notably, we observed consistent UBA3 expression across four ICC cell types, with the highest UBA3 expression identified in HCCC-9810 cells ( Figure 2A,B). Next we detecteed the expression of UBA3 in a cohort of 50 normal liver tissues and 50 ICC tissues by immunohistochemistry assay. We found that the level of UBA3 was significantly higher in ICC tissue than in normal liver tissue ( Figure 2C).

### Knockdown of *UBA3* inhibits the proliferation and migration of ICC

**Figure 3:**
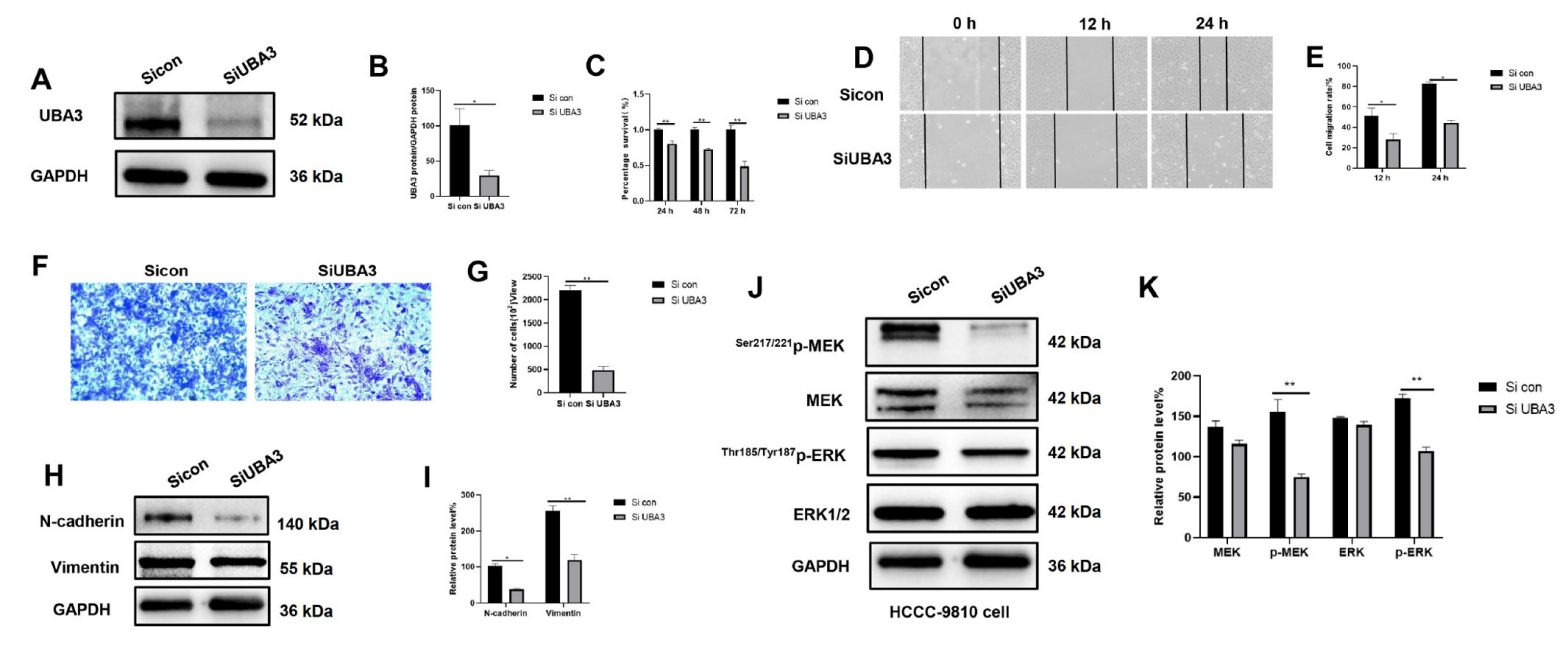
Figure 3 *UBA3* knockdown inhibited the proliferation and migration of ICC (A,B) UBA3 was knocked down in HCCC-9810 cells. (C) In HCCC-9810 cells, proliferation was detected by the CCK8 assay at 24, 48 and 72 h. (D,E) The migration of HCCC-9810 cells was detected by wound healing assay at 12 and 24 h. Magnification: 100×. (F,G) The migration of HCCC-9810 cells was detected via transwell assay at 24 h. Magnification fold: 100×. (H,I) The expressions of N-cadherin and vimentin in HCCC-9810 cells were detected by western blot analysis at 48 h. (J,K) The expressions of MEK, Ser217/221p-MEK, ERK and Thr185/Tyr187p-ERK protein in HCCC-9810 cells were detected by western blot analysis at 48 h.

As UBA3 expression was relatively high in ICC cells, siRNA control and UBA3 siRNA were transfected into HCCC-9810 cells to knock down *UBA3*. The transfection efficiency was confirmed by western blot analysis ( *P* <0.05; Figure 3A,B). CCK-8 assay showed that knockdown of *UBA3* significantly inhibited the proliferation of HCCC-9810 cells ( *P*<0.01; Figure 3C). The results of the wound healing assay showed that *UBA3*-knockdown HCCC-9810 cells migrated more slowly than control cells at 12 and 24 h ( *P*<0.05; Figure 3D,E). We then performed transwell assay to further analyze the effect of UBA3 on the migration of HCCC-9810 cells. The results showed that knockdown of *UBA3* significantly reduced the number of migrating cells ( *P*<0.01; Figure 3F,G). The expression of N-cadherin and vimentin was inhibited after *UBA3* was knocked down, as detected by western blot analysis, suggesting that *UBA3* is an oncogene in ICC ( Figure 3H,I). These results suggest the substantial inhibitory effects of *UBA3* knockdown on ICC progression and the potential therapeutic value of UBA3 intervention.

### *UBA3* knockdown inhibits the MAPK signaling pathway in ICC cells

Mitogen-activated protein kinase (MAPK) is an important signaling pathway that regulates a variety of biological processes. ERK expression is vital for cancer development, and its overactivation promotes cell proliferation, invasion and migration. The MEK/ERK pathway is the most important signaling cascade in the MAPK signaling pathway and plays a crucial role in the survival and development of tumor cells [33]. GSEA predicted the correlation of UBA3 with cell proliferation and MAPK signaling pathway activity. After further verifying the important role of the MAPK pathway in the tumor suppressive effect induced by *UBA3* knockdown, we found that the expression levels of MEK and ERK remained unchanged after *UBA3* knockdown, while the expression levels of ^Ser217/221^p-MEK and ^Thr185/Tyr187^p-ERK were decreased ( Figure 3J,K).

### UBA3 promotes ICC proliferation and migration through ANXA2 activation of the MAPK signalling pathway

**Figure 4:**
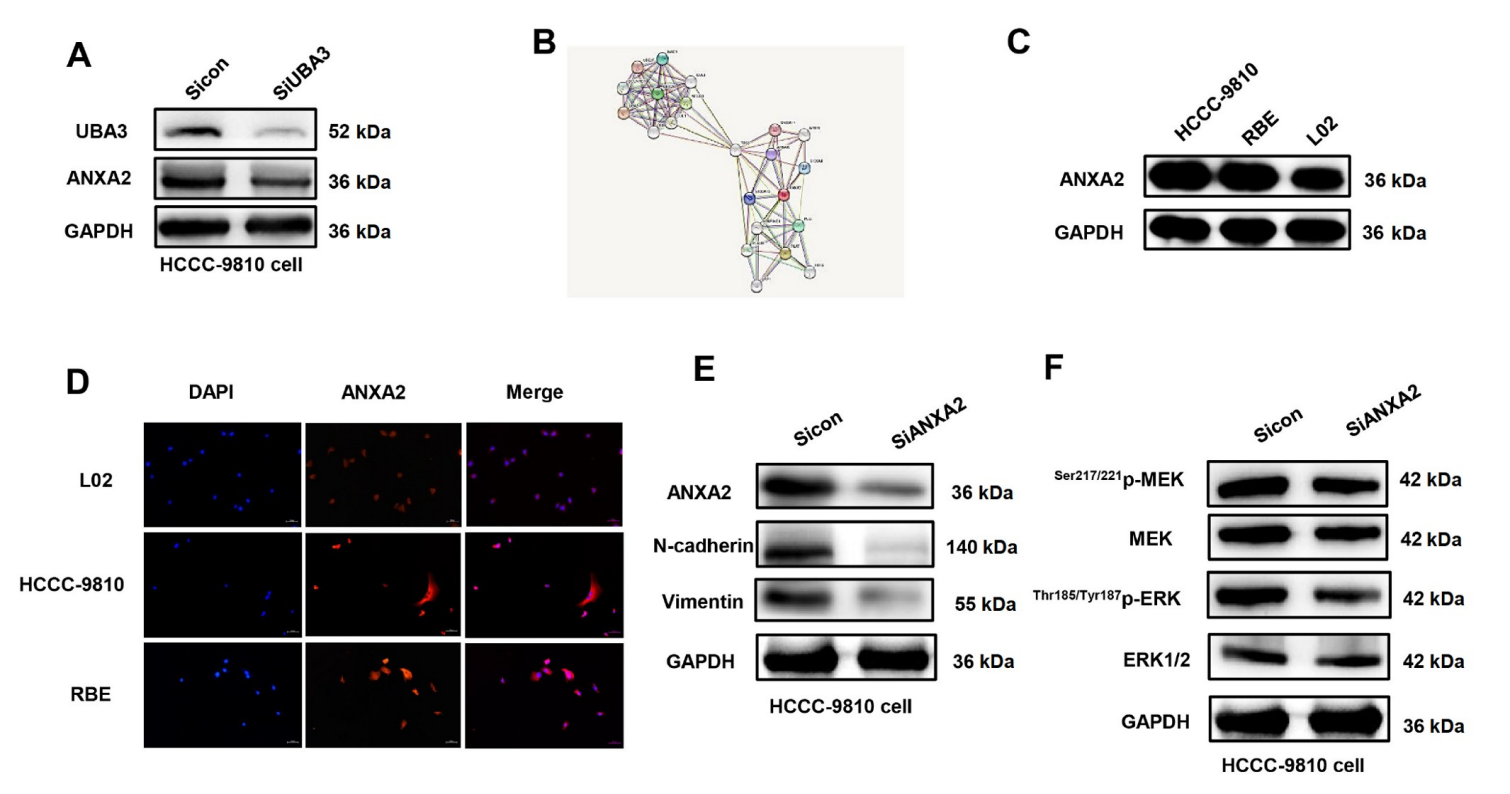
Figure 4 UBA3 promoted ICC proliferation and migration through ANXA2 (A) After siUBA3 treatment, ANXA2 expression was decreased in HCCC-9810 cells. (B) The mediating effect indicates that UBA3 affects ANXA2 via TP53. (C) ANXA2 was highly expressed in ICC cells. (D) ANXA2 is highly expressed in ICC cells and localized in the cytoplasm. Magnification fold: 100×. (E) N-cadherin and vimentin levels were decreased after ANXA2 was knocked down. (F) The protein expressions of MEK, Ser217/221p-MEK, ERK and Thr185/Tyr187p-ERK in HCCC-9810 cells were detected by western blot analysis at 48 h.

The expression of ANXA2 was also reduced after treatment with siUBA3 ( Figure 4A). The presence of a mediating effect indicated that UBA3 and ANXA2 were associated with TP53 ( Figure 4B). Moreover, ANXA2 was highly expressed in HCCC-9810 and RBE cells relative to that in normal L02 hepatocytes ( Figure 4C). In addition, ANXA2 was localized in the cytoplasm, and the fluorescence intensity was greater in HCCC-9810 and RBE cells than in L02 cells, further supporting the high expression of ANXA2 in ICC ( Figure 4D). After siANXA2 treatment, N-cadherin and vimentin expressions were decreased in a dose-dependent manner ( Figure 4E). In addition, the expression levels of MEK and ERK did not change after *ANXA2* knockdown, while the expression levels of ^Ser217/221^p-MEK and ^Thr185/Tyr187^p-ERK were decreased ( Figure 4F).

### Knockdown of *ANXA2* did not affect the proliferative and migratory effects of *UBA3* silencing on ICC

**Figure 5:**
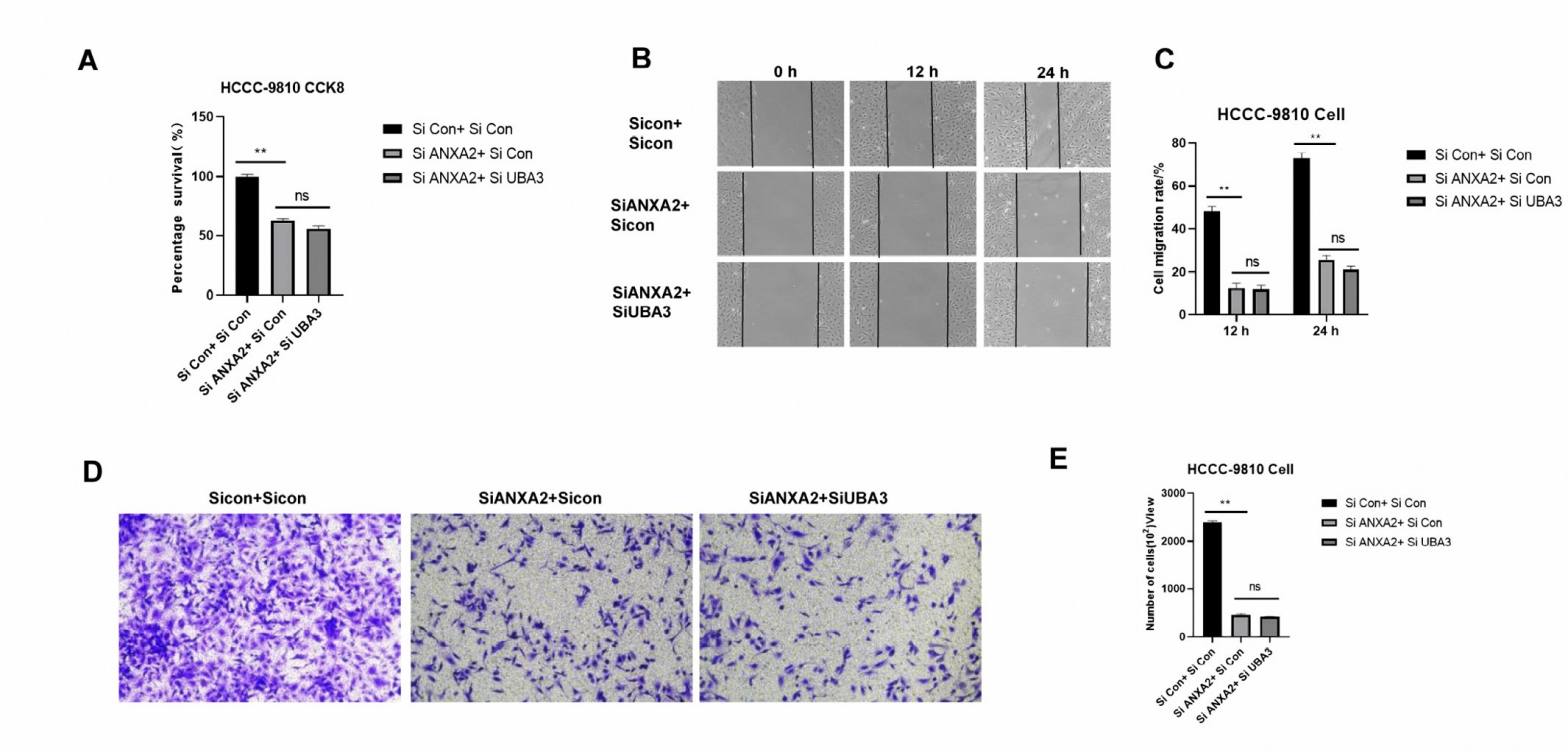
Figure 5 Knockdown of *ANXA2* did not affect the proliferative and migratory effects of *UBA3* silencing on ICC (A,B) The migration of HCCC-9810 cells was detected by wound healing assay at 12 and 24 h. Magnification: 100×. (C) In HCCC-9810 cells, proliferation was detected by the CCK-8 assay at 72 h. (D,E) The migration of HCCC-9810 cells was detected via transwell assay at 48 h. Magnification: 100×.

To explore that whether knockdown of *ANXA2* affect the proliferative and migratory effects on ICC, we first knocked down *ANXA2* in ICC, and then knocked down *UBA3*. CCK8 ( Figure 5A), wound healing ( Figure 5B,C), and transwell assays ( Figure 5D,E) were performed to detect whether there was a difference in the proliferative and migratory abilities of ICC in the *ANXA2*-silenced group and the group with double knockdown of *ANXA2* and *UBA3*. The results showed that the group with both *ANXA2* and *UBA3* knockdown did not significantly inhibit ICC proliferation or migration compared to the group with *ANXA2* knockdown only. These findings suggest a correlation between UBA3 and ANXA2 in ICC cells.

### UBA3 is a direct target of bufalin

**Figure 6:**
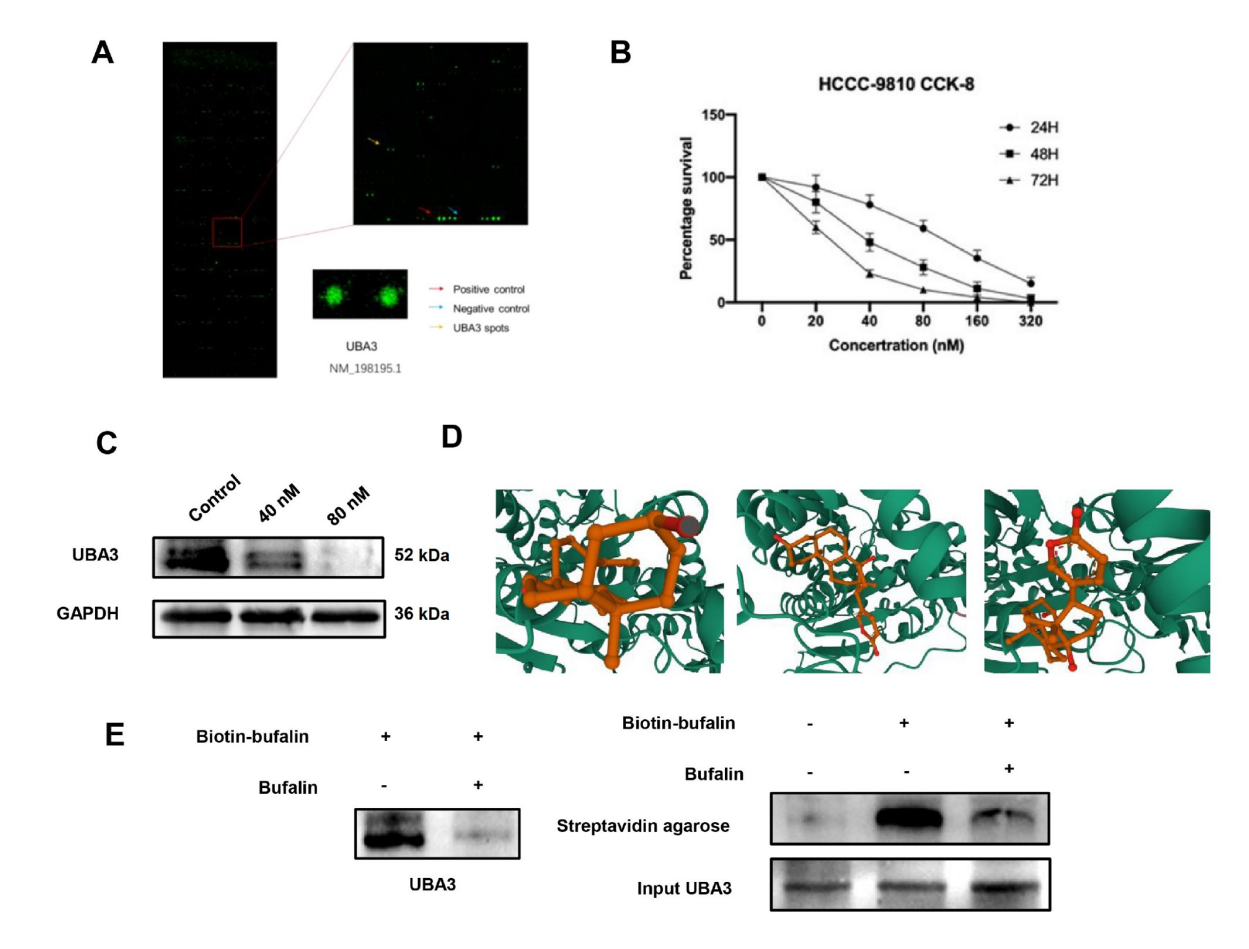
Figure 6 UBA3 is a direct binding target of bufalin (A) Protein microarray showed that UBA3 is the target of bufalin. (B) CCK8 assay showed that bufalin inhibited ICC cell viability in a time and dose-dependent manner. (C) The expression of UBA3 protein was decreased when HCCC-9810 cells were treated with 40 and 80 nM bufalin. (D) The UBA3 protein is docked with the bufalin molecule. (E) Pull-down assay.

To explore the interacting protein, we performed human proteome microarray screening. Among the candidates, UBA3 is strongly associated with bufalin (SNR=1.33; Figure 6A). CCK-8 assay was used to determine the effect of bufalin on HCCC-9810 cells. The results showed that bufalin inhibited ICC cell viability in a time- and dose-dependent manner. The IC _50_ was 80 nM after 24 h and 40 nM after 48 h ( Figure 6B). We found that the expression of UBA3 protein decreased with the increase of bufalin concentration ( Figure 6C). The affinity of bufalin for UBA3 was assessed. The results showed that bufalin binds to UBA3 targets through visible hydrogen bonds and strong electrostatic interactions. In addition, the hydrophobic pocket of each target was successfully occupied by bufalin. The top three sites with low binding energies of –8.160, –7.869 and –7.793 indicated that the binding between UBA3 and bufalin was highly stable ( Figure 6D). To confirm these interactions, we used a pull-down assay, a widely used experimental method for validating protein‒protein interactions. A strong association was detected between UBA3 and bufalin ( Figure 6E), which indicated that UBA3 is a direct interacting target of bufalin.

### Similar inhibitory effects on ICC by bufalin and *UBA3* silencing

**Figure 7:**
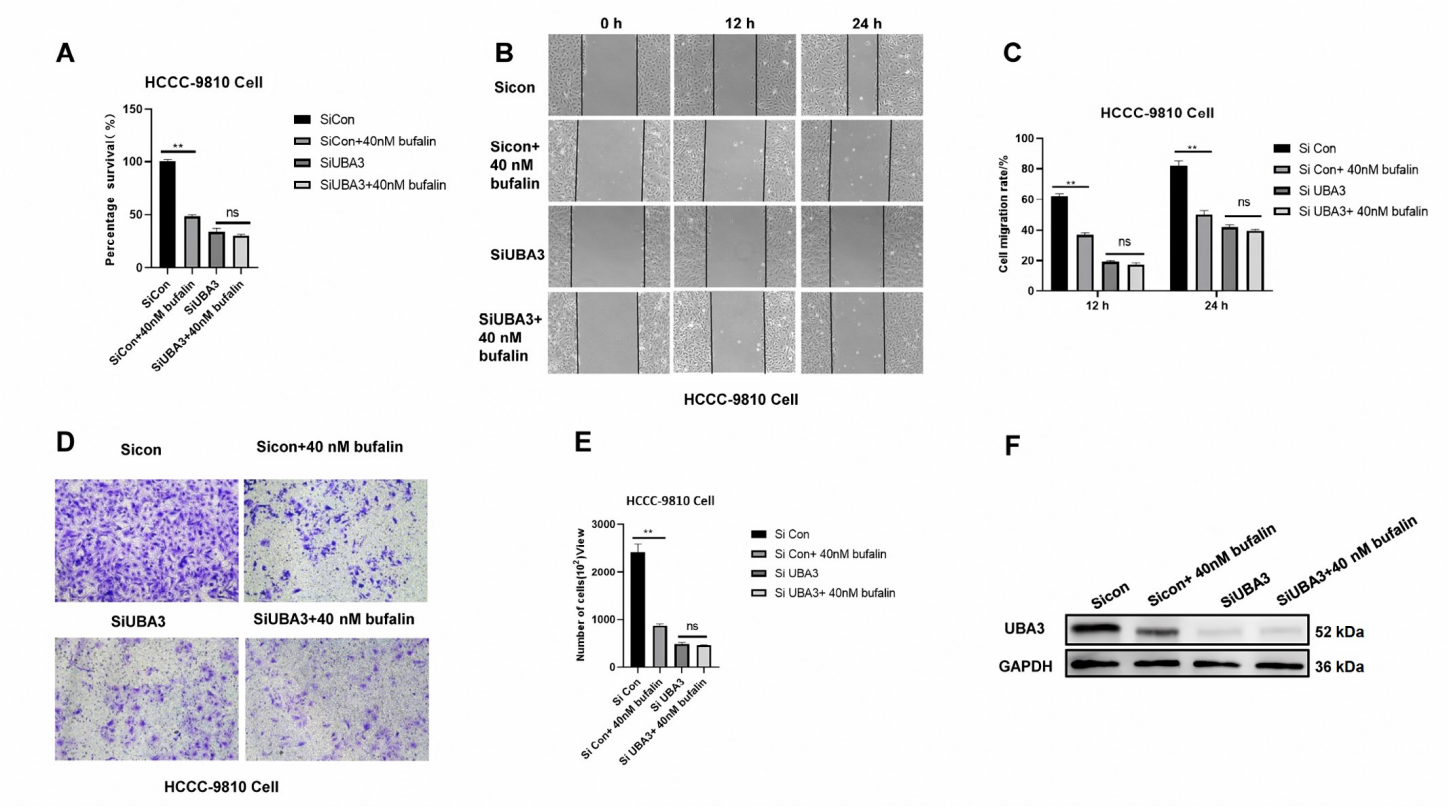
Figure 7 Similar inhibitory effects on ICC by bufalin and *UBA3* silencing (A) In HCCC-9810 cells, proliferation was detected by CCK-8 assay at 72 h. (B,C) The migration of HCCC-9810 cells was detected by wound healing assay at 12 h and 24 h. Magnification: 100×. (D,E) The migration of HCCC-9810 cells was detected via transwell assay at 48 h. Magnification fold: 100×. (F) Western blot analysis of UBA3 expression in HCCC-9810 cells after UBA3 knockdown, or UBA3 knockdown combined with bufalin treatment.

**Figure 8:**
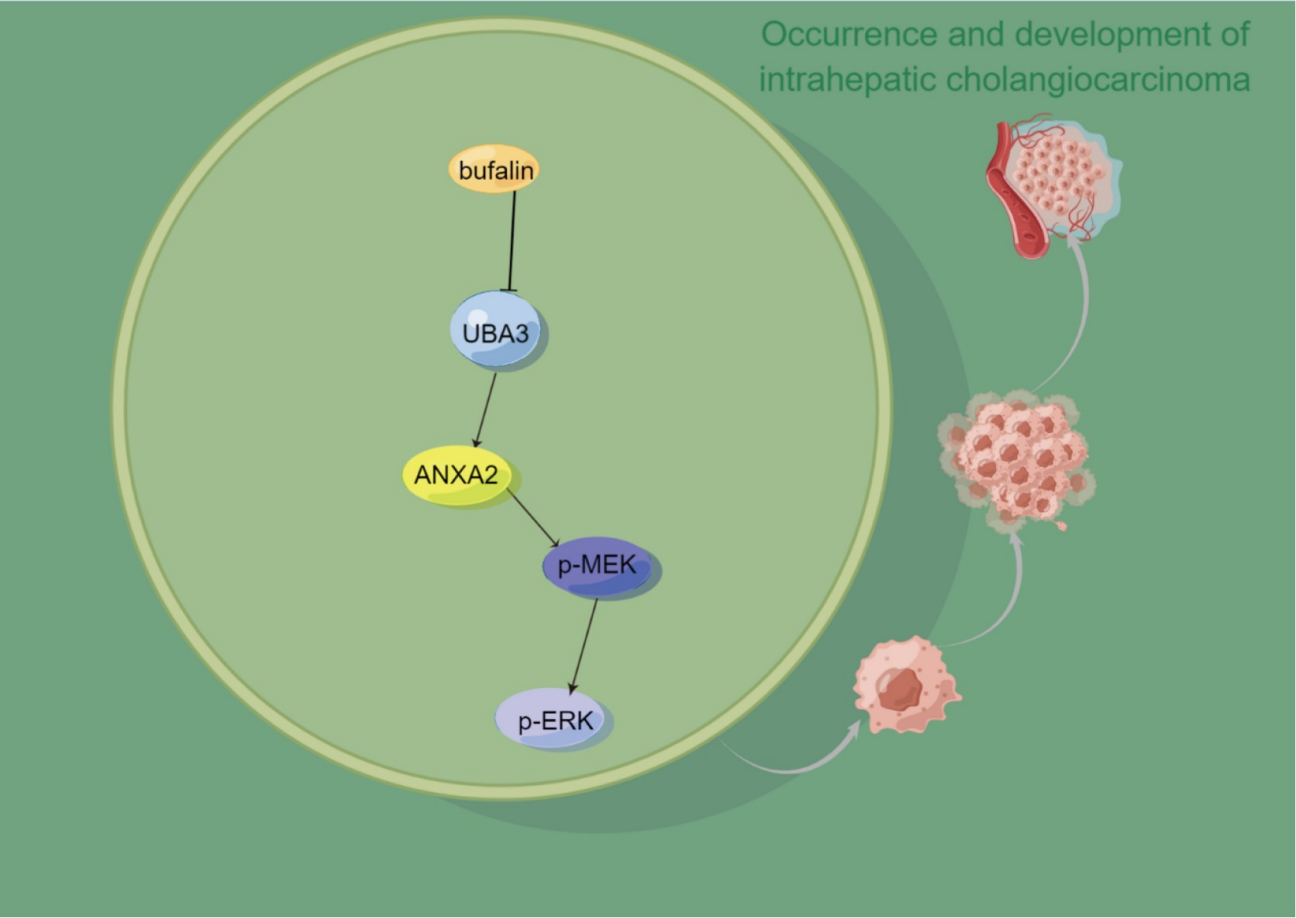
Figure 8 Schematic diagram of UBA3 promotes the occurrence and metastasis of ICC UBA3 is a target of bufalin, and UBA3 activates the expression level of ANXA2, thereby activating the expression levels of p-MEK and p-ERK to promote ICC proliferation and migration. The diagram is drawn using Figdraw software (www.figdraw.com).

To elucidate whether *UBA3* knockdown affects the effects of bufalin on ICC proliferation and migration, we conducted experiments involving *UBA3* knockdown followed by bufalin treatment. CCK-8 ( Figure 7A), wound healing ( Figure 7B,C) and transwell assays ( Figure 7D,E) showed that there was no significant effect on proliferation and migration of HCCC-9810 cells at 12 and 24 h relative to 0 h in the *UBA3* knockdown and *UBA3* knockdown combined with bufalin treatment groups. Western blot analysis showed that bufalin caused a decrease in UBA3 expression. Silencing of *UBA3* in combination with bufalin treatment resulted in no expression of UBA3. ( Figure 7F). Collectively, these results suggest that UBA3 is a target of bufalin and that ICC cells with *UBA3* knockdown are less sensitive to the inhibitory effects of bufalin on proliferation and migration. Finally, the whole results is shown in a schematic diagram ( Figure 8).

## Discussion

In this study, we identified *UBA3* as a signature gene for ICC diagnosis and prognosis. We also revealed the role of UBA3 in ICC proliferation and migration through the MAPK signaling pathway and revealed ANXA2 as a downstream molecule of UBA3. We also screened bufalin as a candidate inhibitor of UBA3 and demonstrated its anti-ICC activity *in vitro*. Our findings provide new insights into the biological functions of UBA3 in ICC and suggest novel strategies for ICC diagnosis and treatment. ICC is one of the most malignant tumors. With the continuous development of medical technology and the deepening of tumor molecular biology research, targeted therapy for ICC is being developed rapidly. The confirmed common mutated genes include ANXA10 [34], CD90 [35], EIF5A2 [36], and HSPB8 [37]. Despite that many potential therapeutic targets have been found, the morbidity and mortality of ICC patients are still increasing, and the overall survival rate is still unsatisfactory; therefore, it is crucial to continue to explore the underlying molecular mechanisms and oncogenes of ICC to provide new ideas for its diagnosis and treatment [ 38, 39].

UBA3 is the catalytic subunit of NAE and initiates neddylation, a posttranslational modification that regulates various cellular processes. UBA3 has been implicated in various biological processes and diseases, such as adipogenesis, oxidative phosphorylation, glucose metabolism, and cancer. However, the role of UBA3 in ICC has not been explored. By using multiple bioinformatics approaches, we selected *UBA3* as a signature gene for ICC diagnosis from the GEO dataset. We found that UBA3 was overexpressed in ICC tissues compared to normal liver tissues and that its expression was positively correlated with tumor size, stage, and grade. We also found that high UBA3 expression was associated with poor overall survival and disease-free survival in ICC patients. As a specific inhibitor of the NEDD8 activation enzyme E1, MLN4924 can bind to the UBA3 subunit at the activation site of E1 to form a covalent ligand, resulting in a loss of E1 enzyme activity and subsequent blockade of the neddylation pathway [ 13, 40]. UBA3 and neddylation promote the growth, survival and cell cycle progression of APL cells and maintain an undifferentiated state [15]. These results suggest that UBA3 is a potential biomarker for ICC diagnosis and prognosis.

Our study demonstrates that UBA3 is highly expressed in ICC, and UBA3 affects ANXA2 through the MAPK signaling pathway to promote ICC proliferation, invasion and migration. UBA3 is a target of bufalin, and bufalin targeting UBA3 inhibits the development and progression of ICC through the MAPK signaling pathway. Nevertheless, our study still has several limitations that need to be addressed in future research. First, our study was based on bioinformatics analysis and *in vitro* experiments. The clinical relevance and applicability of our findings need to be validated in larger and more diverse cohorts of ICC patients and *in vivo* animal models. Second, our study focused on the role of UBA3 in ICC cell proliferation and migration but did not explore its effects on other aspects, such as apoptosis, autophagy, angiogenesis, and the immune response. The molecular mechanisms underlying the regulation of UBA3 expression and activity in ICC also need to be further elucidated. Third, our study screened bufalin as a candidate inhibitor of UBA3 but did not evaluate its pharmacokinetic or pharmacodynamic properties, toxicity or side effects, or synergistic or antagonistic effects with other anti-ICC agents. The optimal dose, route, and schedule of bufalin administration for ICC treatment also need to be determined.

In addition, in the present study, samples with high ICC gene expression were enriched for nuclear translocation, protein localization, the glucagon signaling pathway, apoptosis, the Wnt signaling pathway and other biological processes that contribute to tumor progression. Related literature also indicates that Tbata is expressed in thymic stromal cells and interacts with UBA3, thereby inhibiting the NEDD8 pathway and cell proliferation. Thus, UBA3 may promote ICC proliferation and migration through other processes such as the inhibition of immune cell proliferation [41].

In conclusion, this study presented a comprehensive exploration into the multifaceted role of UBA3 in the context of ICC. Using bioinformatics methods, we identified UBA3 as a potential biomarker for early ICC diagnosis. The robust analysis of UBA3 expression in patient samples underscored the clinical relevance of our findings, indicating that UBA3 may be a promising diagnostic target. Furthermore, our investigation delved into the interplay between *UBA3* knockdown, ICC proliferation, and migration, elucidating its involvement in the MAPK signaling pathway. Notably, UBA3 emerges as a target of bufalin, exerting its inhibitory effects on ICC proliferation and migration by suppressing the MAPK pathway through ANXA2. Inhibition of the proliferation and migration of ICC is an important mechanism for the therapeutic effect of UBA3.

## Supporting information

Supplementary_Figures_final

Supplementary_Figures_final

## Supplementary Data

Supplementary data is available at *Acta Biochimica et Biophysica Sinica* online.
