# Supplementary material for: UBA3 promotes the occurrence and metastasis of intrahepatic cholangiocarcinoma through MAPK signaling pathway: UBA3 promotes the occurrence and metastasis of ICC
Source: Acta Biochim Biophys Sin (Shanghai). 2024 Jan 31;56(2):199–209. doi: 10.3724/abbs.2024014 (PMC10984854; doi:10.3724/abbs.2024014)
Supplement: Supplementary_Figures_final [file Supplementary_Figures_final.pdf]

Supplementary Figures

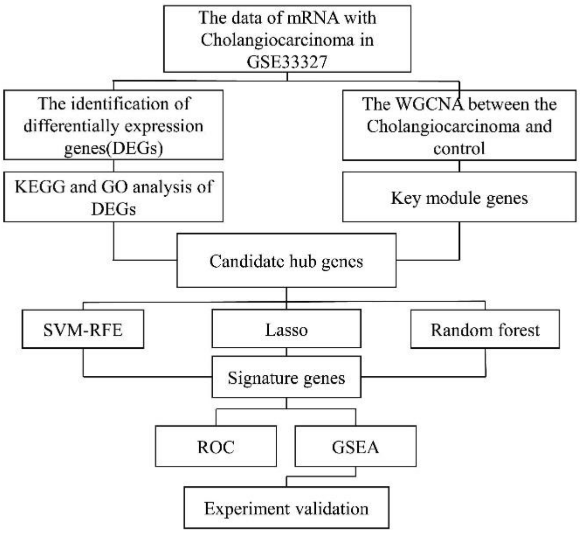

Supplementary Figure S1. The flow chart of this research

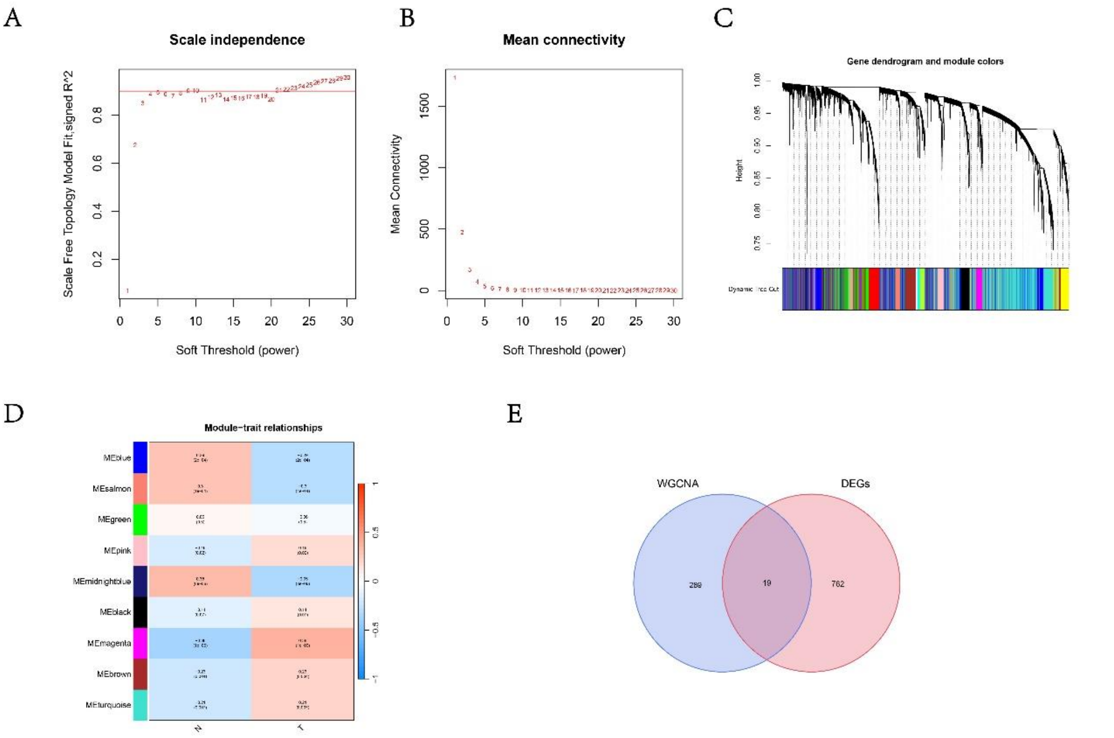

Supplementary Figure S2. WGCNA and identification of hub candidate genes in GSE33327 (A) WGCNA soft threshold power. (B) Linkage of the mean WGCNA. (C) Dendrogram of WGCNA clustering. (D) WGCNA's clustering module. (E) Linked graph showing the interactions of DEGs with genes in the magenta and

midnightbluem modules.

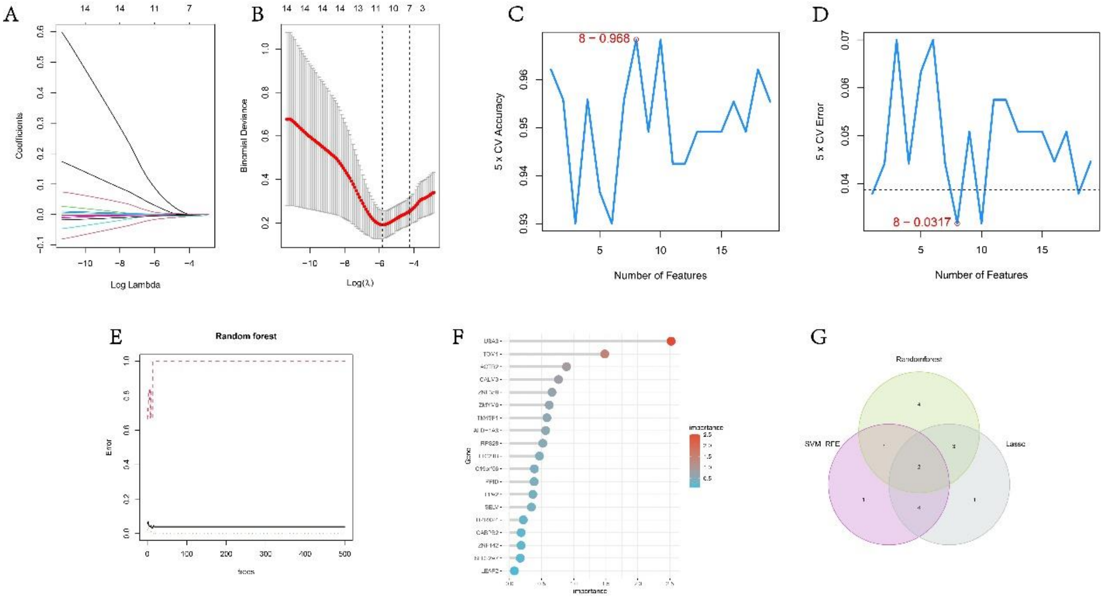

Supplementary Figure S3. The signature genes are analyzed using machine learning algorithm (A) LASSO model penalty plot with error bars indicating standard errors. (B) The scale of change in the coefficient size of the parameters shrinks as the value of the k penalty increases, as shown in the LASSO plot. (C,D) Screening of significant genes based on the SVM-RFE algorithm. (E) Error rate confidence interval of the random forest model. (F) Random forest model with a relative importance of genes greater than 0.25. (G) The interactions of LASSO, SVM-RFE and the random forest algorithm

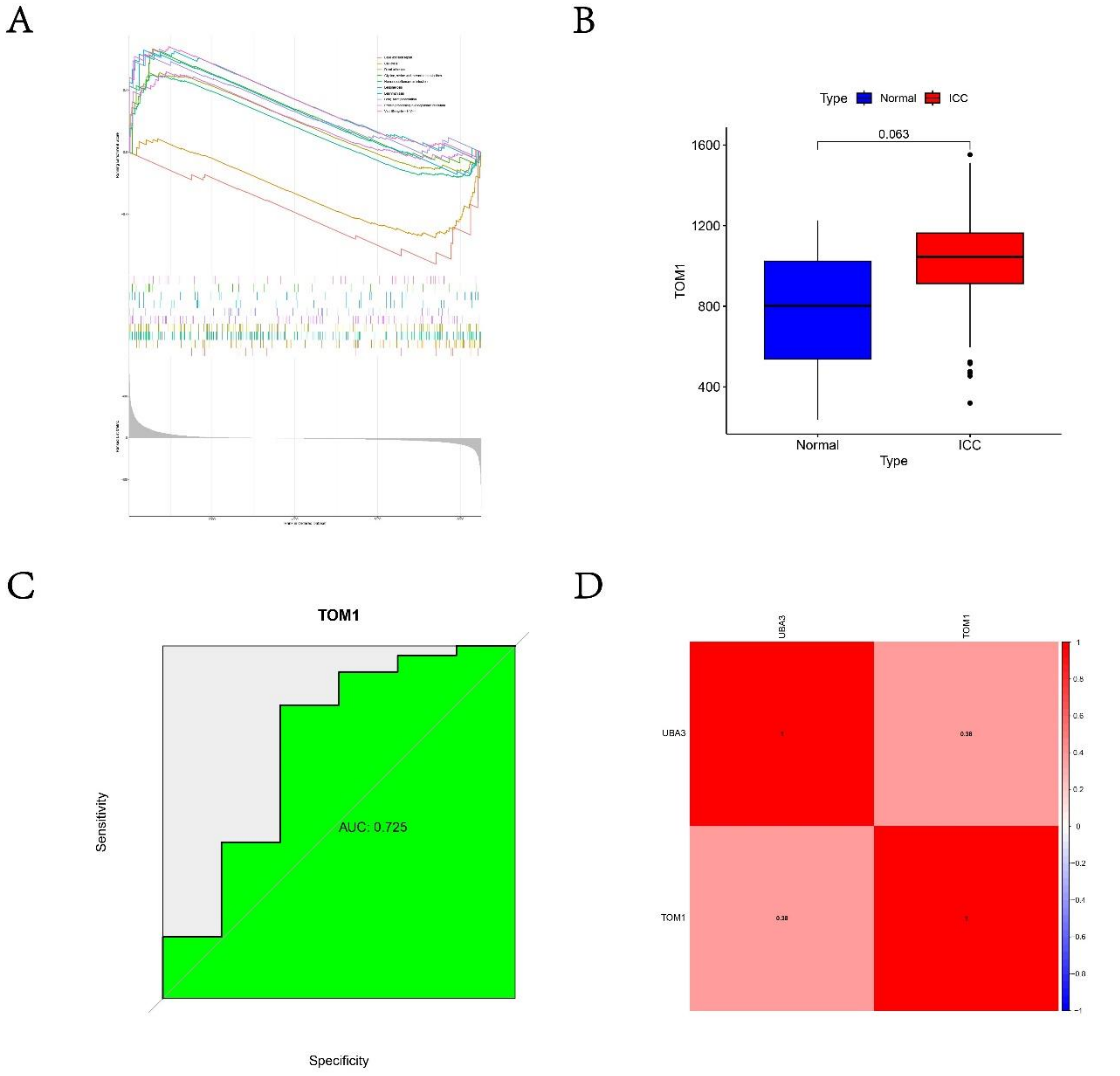

Supplementary Figure S4. GSEA of the signature genes in pediatric patients (A) GSEA of TOM1 in ICC. (B) Correlation between UAB3 and TOM1. (C) TOM1 expression between ICC patients and healthy individuals. (D) Diagnostic performance of TOM1 as shown by the ROC curve.

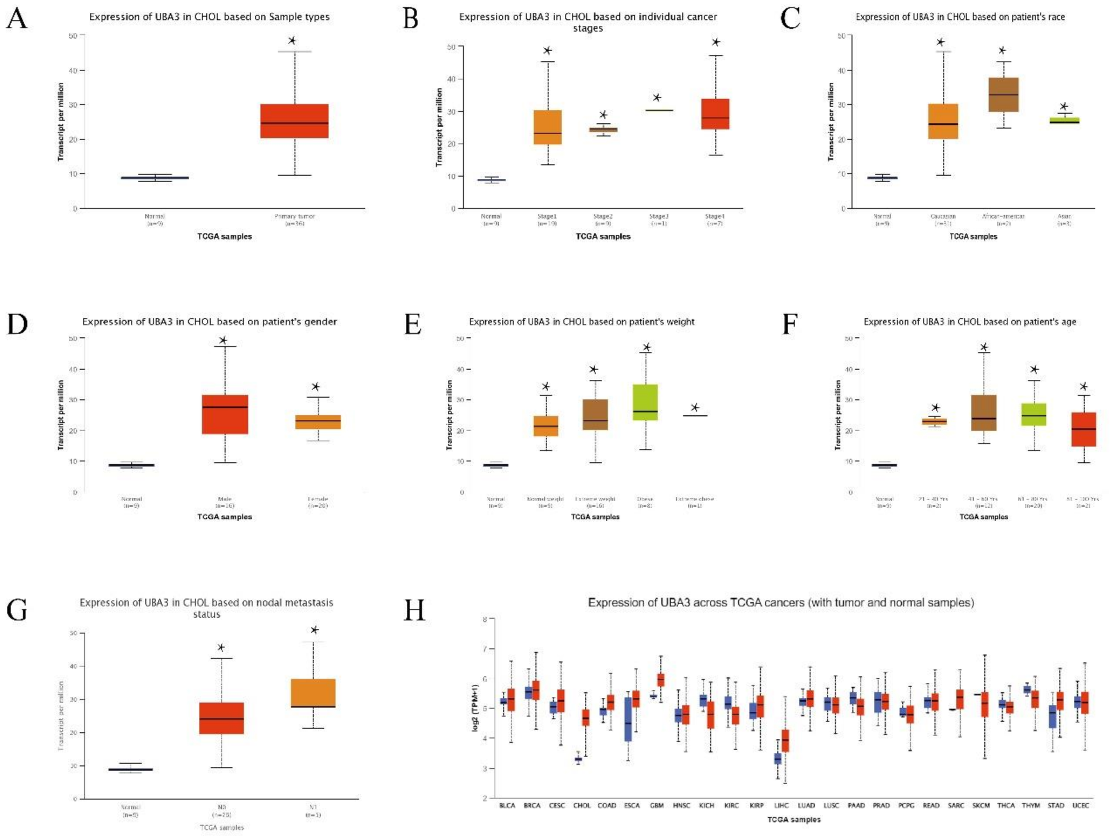

Supplementary Figure S5. Expressions of UBA3 in different sample types (A) Individual cancer stage; (B) Patient race; (C) Patient sex; (D) Patient weight; (E) Patient age; (F) Nodal metastasis status; and (G) Expression of UBA3 in different cancers. (H) UBA3 is highly expressed in ICC.
